# Explainability in transformer models for functional genomics

**DOI:** 10.1093/bib/bbab060

**Published:** 2021-04-08

**Authors:** Jim Clauwaert, Gerben Menschaert, Willem Waegeman

**Affiliations:** Department of Data Analysis and Mathematical Modelling, Ghent University, Coupure Links 653, 9000 Gent, Belgium; Department of Data Analysis and Mathematical Modelling, Ghent University, Coupure Links 653, 9000 Gent, Belgium; Department of Data Analysis and Mathematical Modelling, Ghent University, Coupure Links 653, 9000 Gent, Belgium

**Keywords:** interpretable neural networks, transformers, functional genomics, DNA-binding sites

## Abstract

The effectiveness of deep learning methods can be largely attributed to the automated extraction of relevant features from raw data. In the field of functional genomics, this generally concerns the automatic selection of relevant nucleotide motifs from DNA sequences. To benefit from automated learning methods, new strategies are required that unveil the decision-making process of trained models. In this paper, we present a new approach that has been successful in gathering insights on the transcription process in *Escherichia coli*. This work builds upon a transformer-based neural network framework designed for prokaryotic genome annotation purposes. We find that the majority of subunits (attention heads) of the model are specialized towards identifying transcription factors and are able to successfully characterize both their binding sites and consensus sequences, uncovering both well-known and potentially novel elements involved in the initiation of the transcription process. With the specialization of the attention heads occurring automatically, we believe transformer models to be of high interest towards the creation of explainable neural networks in this field.

## Introduction

Deep learning techniques are increasingly obtaining state-of-the-art performances for a multitude of prediction tasks in genomics [8]. Capable of automating the feature extraction process during training, these techniques flip the conventional approach of machine learning studies; instead of requiring meaningful descriptors that function as input to the model, raw input data is used. As such, identifying biologically meaningful descriptors and their relation to the target label as learned by the model requires a new set of approaches.

Deep learning has offered solutions for functional genomics where the current understanding of biological processes is insufficient [1, 2, 6]. As such, explainable models that can offer insights into the biological process for which the model is trained are likely to play a role in the discovery of new regulatory mechanisms.

Today, the main contributions towards dissecting the workings of neural networks are achieved by mapping the importance of input features towards the model output, either through investigation of the gradient [34] or permutation strategies of the input [41]. These techniques are limited to the evaluation of the input features on the model output and can therefore be limiting when trying to capture the complex mechanisms between multiple regulatory elements involved in biological processes.

In a previous study, we introduced a model for performing annotation tasks on the genomic sequences of prokaryotes, achieving state-of-the-art results for the identification of transcription start sites (TSSs), translation initiation sites and methylation sites. This model is based on the transformer-XL architecture, first introduced for natural language processing [7]. Vaswani $et$$al$. [38] found that the attention heads, specialized units making up the core mechanism of the neural network, could map relations that convey semantic meaning. Based on this finding, we aim to investigate new ways of extracting biological meaning from a transformer network trained on annotating the genome.

This work offers several contributions: (i) we evaluate and compare the annotations of a variety of recent, mostly sequencing based, *in vivo* methodologies for the detection of TSSs applied on *Escherichia coli*. Based on four independent data sets, we curate an improved set of annotations; (ii) we lay out our approach to characterize the function of attention heads. Many of these are specialized towards detecting the presence of regulatory elements based on sequence and positional information; (iii) using this information, we offer a comparison with existing knowledge on the mechanisms involved during the transcription process. As such, we link existing findings to literature and discuss their relevance; and (iv) using the model output predictions along the full genome sequence, we get a better understanding into the model characteristics and potential flaws. Based on these results, we conclude that the analysis of the discussed models for genome annotation tasks offer singular potential for extracting biological meaning, where the discussed techniques are applicable for any genome annotation task.

## Related work

The creation of methodologies to facilitate the interpretation and role of nucleotide sequences started with mathematical formulas for consensus sequences [13], weight matrices [35] and rank alignments [20, 42]. An increasing pool of understanding on molecular biology gave rise to meaningful descriptors of nucleotide sequences, such as GC content, bendability [22], flexibility [3] and free energy [19]. The recent publication of Seq2Feature, which uses 41 descriptors to characterize nucleotide sequences, indicates that meaningful sequence descriptors are still relevant today [24].

Despite efforts towards creating meaningful descriptors of nucleotide sequences, the understanding of how these influence biological processes is still lacking. Even with the help of early machine learning methods, the role of DNA for many biological processes is not properly understood. Today, the branch of deep learning is increasingly returning state-of-the-art performances for a variety of tasks in the field of functional genomics [44]. By integrating the feature extraction process during training, models are capable to automatically map relations from the raw DNA sequence without the need of high-level descriptors. However, in contrast to earlier applications, a look into the decision-making process of the trained network is required to gain understanding of the relation between the nucleotide sequence and biological process.

Today, two popular strategies exist that aim to offer insights into the workings of neural networks by evaluating the influence of the input features towards the output prediction. The 1st group achieves this by targeted permutation of the input features, first described by Zeiler $et$$al$. [41]. A multitude of other strategies have also been described, including the work of Fisher $et$$al$.[11] and Zintgraf $et$$al$. [43]. The other group of approaches seeks to investigate the partial derivatives of the model output class with respect to the input features. The technique was first discussed by Simonyan $et$$al$. [34] in 2014 and has been built upon for the construction of a variety of tools thereafter [32, 36]. In the combined fields of genomics and deep learning, these ideas have been borrowed to map important sites and nucleotides motifs. For example, Alipanahi $et$$al$. [1] perform a sensitivity analysis to guide the construction of motifs for protein-binding sites on the DNA. The sensitivity of nucleotides has been evaluated towards the methylation state by Angermueller $et$$al$. [2]. Hill $et$$al$. [15] permute input sequences to investigate the influence on translation initiation and termination sites. By applying a pairwise mutation map, the correlation of two positional features is shown. Another study has leveraged the analysis of partial derivatives for the construction of motifs around splice sites in eukaryotes [45].

## Methods

### Data sources

To reduce the potential influence of noise, the annotations from a variety of recent, high-precision *in vivo* experimental methods were compared for the detection and annotation of TSSs in prokaryotes: Cappable-seq by Etwiller $et$$al$. [9], retrieving 16 348 TSSs, Single-Molecule Real-Time Cappable-seq (SMRT-Cappable-seq), by Yan $et$$al$., [40], retrieving 2311 TSSs and Simultaneous 5*′*- and 3*′*-end sequencing (SEnd-seq), by Ju $et$$al$. [18], retrieving 4026 TSSs.

Given its importance to the community, TSS listed by RegulonDB has also been included. RegulonDB features an up-to-date collection of manually curated and automated (differential RNA-seq) TSSs from a plethora of independent sources [29]. It has been the chosen data set for several recent machine learning tasks aimed at identifying TSSs [21, 27, 39]. The positive set consists of the positions marked with ‘strong evidence’, summing to a total of 6487 annotated positions. An overview of the data is listed in Table 1.

**Table 1:** Overview of the data sets properties used in this study $Notes$: From left to right: data sets source, *in vivo* next-generation sequencing technique, number of positive and negative labels and the partition of TSS shared with at least one other data sets. The annotations are derived or mapped (Cappable-seq) on *E. coli* MG1655 (accession: ‘NC_000913.3’). The total genome consists of 9 283 304 nucleotides.

Data source	Technique	Year	Positive labels	Negative labels	Shared TSSs
RegulonDB [29]	Collection	–	6487 (0.07%)	9 276 817 (99.93%)	2476 (38.17%)
Etwiller $et$$al$. [9]	Cappable-seq	2016	16 348 (0.18%)	9 266 956 (99.82%)	4631 (28.33%)
Yan $et$$al$. [40]	SMRT-Cappable-seq	2018	2574 (0.03%)	9 280 730 (99.97%)	2311 (89.78%)
Ju $et$$al$. [18]	SEnd-seq	2019	5502 (0.06%)	9 277 802 (99.94%)	4026 (73.17%)

### Model architecture

The model is built from the transformer-XL [7] architecture. In general, transformer models are increasingly replacing recurrent neural networks, as these architectures have shown to be better suited for optimization on sequential data, resulting in improved training times and performances. Our model furthermore includes convolutional layers, typically applied for the detection of local patterns (Supplementary Table A2), and thereby enhance the detection of nucleotide motifs. The applied model has been proposed in a previous work, which includes an in-depth comparison of existing deep learning methods applied for genome annotation tasks [6].

**Figure 1:**
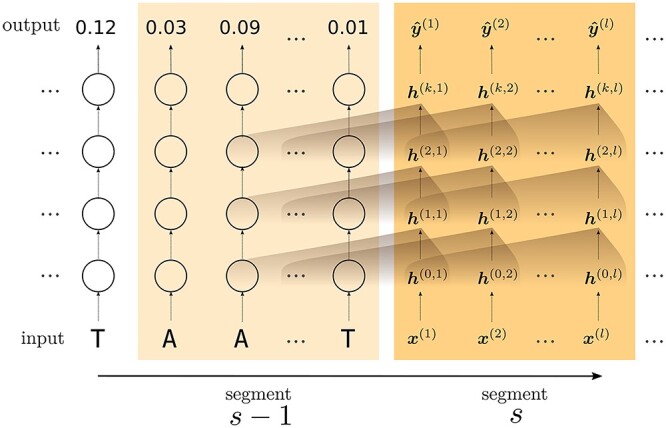
Illustration of the data flow within the transformer architecture. The full genome is processed in sequential segments $s$ with length $l$. First, the input nucleotide is transformed into a vector embedding $\boldsymbol{h}^{(0)}$, after which it is processed by $k$ consecutive residual blocks ($ \boldsymbol{h}^{(0)} \rightarrow \ldots \rightarrow \boldsymbol{h}^{(k)}$). A set of fully connected layers transforms $\boldsymbol{h}^{(k)}$ into a model output $\boldsymbol{\hat{y}}$. For the calculation at each residual block, the upstream $l$ hidden states of the previous layer are applied (brown gradient). For example, the calculation of $\boldsymbol{h}^{(1,l)}$ is based on the hidden states $[\boldsymbol{h}^{(0,1)}, \ldots , \boldsymbol{h}^{(0,l)}$]. Hidden states from the previous segment ($s-1$) are made accessible for the calculation of the hidden states in segment $s$.

The full genome sequence is processed in consecutive segments of length $l$. Every input nucleotide $x \in \{A, C, G, T\}$ is first transformed into a vector embedding $\boldsymbol{h}^{(0)}$, after which it is transformed $k$ times through addition (residual connection) with another vector, obtained by the multi-head attention function present in each layer ($ \boldsymbol{h}^{(0)} \rightarrow \ldots \rightarrow \boldsymbol{h}^{(k)}$). A set of fully connected layers transforms $\boldsymbol{h}^{(k)}$ into a model output $\boldsymbol{\hat{y}^{(k)}}$. For each residual block, the vector that is summed with the input (to obtain $\boldsymbol{h}^{(1)}, \ldots , \boldsymbol{h}^{(k)}$) is calculated using the hidden states of $l$ upstream positions. Figure 1 illustrates the relation between intermediary values within the network. If required, hidden states from the previous segment ($s-1$) are accessible for the calculation of the new hidden states in segment $s$.

The multi-head attention applied in each residual block is methodologically identical. From each input hidden state $\boldsymbol{h}$, a query ($\boldsymbol{q}$), key ($\boldsymbol{k}$) and value ($\boldsymbol{v}$) vector of equal shapes are calculated. The output $\boldsymbol{z}$ of the attention head, applied on the hidden state at position $n$, is calculated as follows:

$$\begin{align*} \boldsymbol{z}^{(n)} = \textrm{softmax}(\frac{\boldsymbol{q}^{(n)} \cdot \boldsymbol{K}}{\sqrt{d_{\textrm{ {head}}}}}) \cdot \boldsymbol{V}, \end{align*}$$

where $\boldsymbol{K}$, $\boldsymbol{V} \in \mathbb{R}^{l \times d_{\textrm{{heads}}}}$ are the matrices that are composed from $l$ upstream hidden states (e.g. $\boldsymbol{K} = [\boldsymbol{k}^{(n-l)}, \ldots , \boldsymbol{k}^{(n)}] $). The denominator is used to stabilize the scores based on the dimensions of $\boldsymbol{q}$, $\boldsymbol{k}$ and $\boldsymbol{v}$ ($d_{\textrm{{head}}}$). The multiplication of the query vector with all the key vectors results in a vector of scores that is normalized for all input values using the $\textrm{softmax}$ function. These scores are multiplied to the $\boldsymbol{v}$ vectors for the calculation of $\boldsymbol{z}$ (i.e. a linear combination). Essentially, attention scores denote the relevance of information present between two positions, where the multiplication of the $\boldsymbol{q}$ and $\boldsymbol{k}$ vectors function as a lock and key encoding, which returns goodness-of-fit scores for the information embedded in two hidden states (defined by $\boldsymbol{v}$). In each residual block, multiple attention heads are present (hence, multi-head attention), each featuring their own unique sets of model weights to calculate $\boldsymbol{q}$, $\boldsymbol{k}$ and $\boldsymbol{v}$. As such, multiple types of information can be extracted from the input hidden states. The outcome of different attention heads within the same layer is further processed into a single vector, which is summed with $\boldsymbol{h}$ to obtain the hidden state of the next layer (e.g. $\boldsymbol{h}^{(1)} \rightarrow \boldsymbol{h}^{(2)}$). A detailed overview of the mathematical operations is given in Supplementary File 1.

In a previous study, we found that the contextual information embedded within the hidden states derived from single nucleotides is limited. Motifs formed from multiple neighboring nucleotides are generally deemed of greater importance towards biological processes. The addition of a convolutional layer allows the $\boldsymbol{q}$, $\boldsymbol{k}$ and $\boldsymbol{v}$ vectors to be derived from multiple neighboring hidden states without affecting the input/output resolution. Thereby, the retrieval of relevant information using attention is improved, resulting in improved predictive performances on a variety of tasks [6].

Positional information is required within the vectors $\boldsymbol{q}$, $\boldsymbol{k}$ and $\boldsymbol{v}$, as these are not implicit through the architecture of the network. This is achieved by superimposing (i.e. through summation) a positional encoding vector to $\boldsymbol{h}$. The added signal is a function of the vector index and the relative positioning with respect to the other input hidden states. It was shown that the transformer model is able to extract this information well and use it to obtain better performances [7, 38].

A model architecture was optimized for the detection of TSSs in a previous study, resulting in a model with a segment length of 512 nucleotides, six layers (i.e. residual blocks) and six attention heads within each residual block [6]. To include information downstream of the annotated TSS towards its annotation, labels can be shifted downstream during processing times in order to include this information (i.e. position upstream). In accordance to recent publications on the detection of TSSs, the downstream bound is positioned 20 nucleotides downstream of the TSS. A detailed overview of the model architecture is given in Supplementary File 1.

### Training and evaluation

The transformer-based model was trained using the full genome sequence, resulting in a total sample size of 9 283 204. The sizes of the positive and negative samples are listed in Table 1. As the model iterates over the genome sequentially, the training, test and validation set are created by splitting the genome at three positions that constitute 70, 20 and 10% of the genome, respectively. The training, test and validation sets have equal input and output class distributions. In order to compare all data sets on the same genome, annotations of the Cappable-seq experiment, originally mapped on the ‘U00096.2’ reference genome, have been remapped on the ‘NC_000913.3’ genome. The same genomic regions are used to train and evaluate all models, at positions 2 738 785, 3 667 115 and 4 131 280 (in accordance to the previous study), as indexed by the ‘RefSeq’ genome (accession: ‘NC_000913.3’). Both the sense and antisense components of these strands are thereby included within the same sets to warrant no unforeseen information transfer between the different sets. The minimum loss on the validation set is used to determine the point at which the network training is halted. Given the prediction task to be a binary classification problem, the cross-entropy loss is used. The performance metrics of the models are obtained by evaluation on the test set.

The Area Under the Receiver Operating Characteristics Curve (ROC AUC) and Area Under the Precision Recall Curve (PR AUC) are the used evaluation metrics. ROC AUC performances represent the area under the curve created by connecting the true positive ($y$-axis) and false positive ($x$-axis) rate of the predictions at different thresholds of the output probability. However, due to the imbalance between positive and negative labels, even a small false positive rate can result in set of false positive predictions that heavily outweighs the true positives in absolute count. PR AUC represents the area under the curve created by connecting the precision (precision, $y$-axis) and recall ($x$-axis). The impact of false positive predictions for the PR AUC is, unlike the ROC AUC, not proportional to the size of the negative set. Therefore, this metric gives a better depiction of the model’s ability in a practical setting, where the top-$k$ positive predictions are of interest.

### Model analysis

For the calculation of the model output at position $n$, each attention head calculates attention scores based on the hidden state of $n$ and the 511 ($l$) upstream hidden states in that layer. Specifically, as outlined in Section 3.2, this is achieved by multiplication of the $\boldsymbol{q}$ and $\boldsymbol{k}$ vectors and consecutive normalization using the $\textrm{softmax}$ function. For a model featuring 36 attention heads and a test set of 1 856 660 nucleotide positions (samples), a total of about $3.4 \cdot 10^{13}$ attention scores have been calculated. To reduce the number of values processed, the scores of the upstream hidden states of only a small number of samples is processed. Specifically, in addition to ca. 1% randomly sampled positions of the test set (18 295 samples), we have included grouping the attention scores according to the nucleotide positions, which result in the highest and lowest model output probabilities (500 each), allowing a look into the influence of certain attention heads on the model output and the differences between the profiles of the averaged attention scores.

## Results

### The evaluated annotation sets have many different annotated start sites

**Figure 2:**
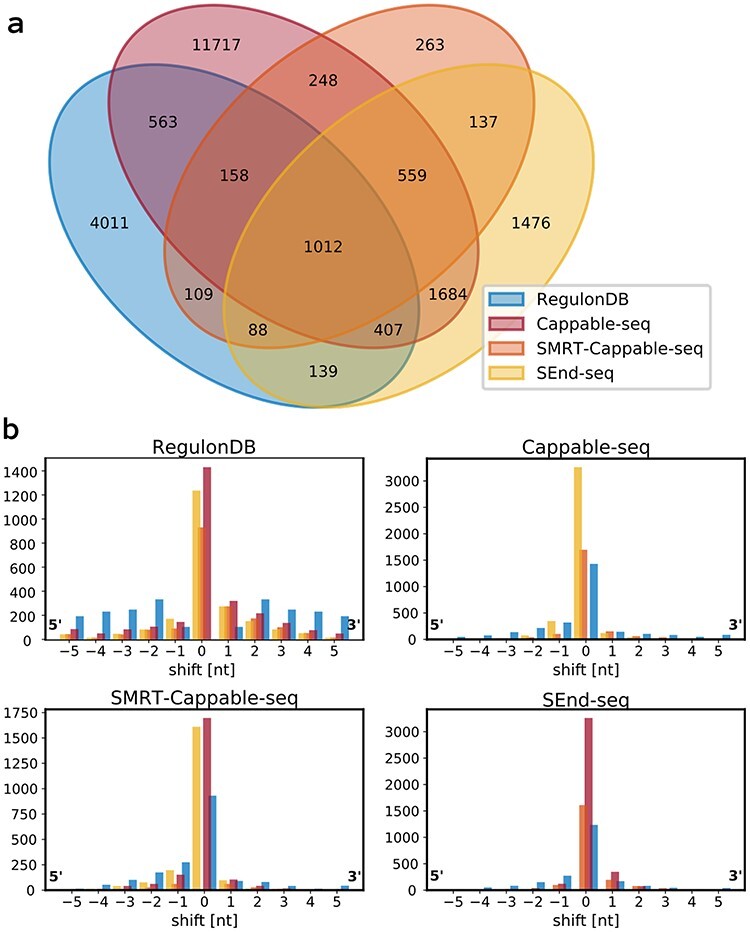
Comparisons between transcription start site (TSS) annotations provided by varying methodologies in *E. coli*. The annotations are obtained from RegulonDB [29] and recent publications introducing novel methodologies from Etwiller $et$$al$. [9] (Cappable-seq), Yan $et$$al$. [40] (SMRT-Cappable-seq) and Ju $et$$al$. [18] (SEnd-seq). (**A**) A venn diagram represents the overlap of annotated TSSs between the different data sources. TSSs are considered to be shared when annotated within five nucleotides of one another. (**B**) The distribution of the annotation shift occurring between the shared TSSs within the five-nucleotide distance used to denote a TSS as shared. Distributions are centered on the annotated position for each data set separately. Within a single data set, it is also possible for multiple TSS to be annotated within close vicinity.

In order to optimize the set of TSS annotations in *E. coli*, a variety of data sets is processed. Yan $et$$al$. [40] consider TSSs between two sets of annotations to be shared if they are positioned within a distance of five nucleotides. According to this criterion, the distribution of shared TSSs between the four data sets is given in Figure 2A. Only 1012 positions are shared between all four data sets. The total number of TSSs shared by at least three and two data sets is 2224 and 5104, respectively. The fraction of TSSs that are listed by any of the other data sets is the least for RegulonDB (38.17%) and Cappable-seq (28.33%). SMRT-Cappable-seq (89.78%) and SEnd-seq (73.17%) feature the highest fraction of shared TSSs (Table 1). However, as the sizes of the data sets differ, percentages should be considered with care. For example, with 16 348 annotations, Cappable-seq features more than twice as many TSSs than any other data set. Figure 2B shows the distributions of the distances between the TSSs within the five nucleotide window used in Figure 2A. The percentage of shared TSSs that are annotated at the exact position constitutes only 52.9, 51.1 and 57.0% for RegulonDB on Cappable-seq, SMRT-Cappable-seq and SEnd-seq, 52.9, 78.7 and 83.4% for Cappable-seq on RegulonDB, SMRT-Cappable-seq and SEnd-seq, 51.1, 78.7 and 77.7% for SMRT-Cappable-seq on RegulonDB, Cappable-seq and SEnd-seq, and 57.0, 83.4 and 77.7% for SEnd-seq on RegulonDB, Cappable-seq and SMRT-Cappable-seq.

Given the differences between annotations present in all data sets, a curated annotation is made that is largely based on the intersection of the discussed data sets rather than the union of the collected annotations. Specifically, the following rules have been applied to include a given TSS to the curated set: (1) the TSS is detected by at least two separate data sets (maximum distance of five nucleotides); (2) the exact position of a shared TSS is determined by the majority vote. In case of a tie, the position is selected based on the novelty of the technique (SEnd-seq > SMRT-Cappable-Seq > Cappable-seq > RegulonDB); (3) finally, given the novelty of SEnd-seq and its high overlap with other data sets, all TSSs detected from this technique have been added. The resulting data set has a total of 6580 positively labeled positions, denoted as the curated set henceforth.

### Improved model performances indicate a better quality of the curated custom set

In order to offer a means of validation about the quality of the annotations and the various steps followed to create the curated set, the performances of different models on the variety of sets have been trained and evaluated. The validation and test sets span identical regions of the genome. Therefore, performances of trained models on the test set can be easily evaluated for different annotations. Table 2 gives a full overview of the performances of all the models on each of the test sets.

**Table 2:** Performance metrics of the transformer network trained and tested on the labeled transcription start sites obtained from RegulonDB [29], Etwiller $et$$al$. [9] (Cap(pable)-seq), Yan $et$$al$. [40] (SMRT-(Cappable-)seq), Ju $et$$al$. [18] (SEnd-seq) and the curated set $Notes$: A model has been trained (rows) and evaluated (columns) on each set of annotations. Both the Area Under the Receiver Operating Characteristics Curve (ROC AUC) and Area Under the Precision Recall Curve (PR AUC) are given for each set-up.

**Train set**	**Test set**
	RegulonDB	Cap-seq	SMRT-seq	SEnd-seq	Custom
**ROC AUC**					
RegulonDB [29]	**0.882**	0.815	0.923	0.882	0.885
Cap-seq [9]	0.790	**0.961**	0.938	0.945	0.945
SMRT-seq [40]	0.749	0.899	0.958	0.961	0.956
SEnd-seq [18]	0.669	0.835	0.944	0.978	0.964
Curated	0.740	0.920	**0.976**	**0.981**	**0.976**
**PR AUC**					
RegulonDB [29]	0.030	0.026	0.053	0.057	0.064
Cap-seq [9]	0.014	**0.132**	0.029	0.039	0.044
SMRT-seq [40]	0.029	0.044	0.086	0.081	0.089
SEnd-seq [18]	0.035	0.052	0.098	**0.128**	0.137
Curated	**0.039**	0.057	**0.141**	**0.128**	**0.141**

The best performances on each test set are given in boldface.

The ROC/PR AUC scores show that the model trained on the curated annotations has the best overall performances on each of the test sets. The highest ROC AUC score on the SMRT-Cappable-seq and SEnd-seq test set is returned by the model trained on the curated TSS annotations. A possible explanation lies in a lower occurrence of false negative predictions in the model’s output due to a reduced number of missing annotations in the training data. False negative predictions come at a higher cost than false positive predictions, as the true positive rate ($y$-axis) is more sensitive to change than the false positive rate ($x$-axis) as an effect of the different sizes of the denominator (i.e. class imbalance).

The low scores of the PR AUC metric illustrate that, despite the high ROC AUC scores, the true positive predictions are outnumbered by the false positive predictions. Even though these scores are in line with the state-of-the-art performances previously reported, application of the model on the full genome still results in models with a low precision/recall ratio.

The overall higher performances of the models trained and tested on the curated annotations provide an affirmation that the combined set of TSSs, obtained through the steps as outlined in the previous section, have better properties than any of the individual data sets. As such, all further results are obtained from the model trained and evaluated on the curated set.

### The majority of attention heads is highly selective towards certain promoter regions

The attention mechanism is used by the model to selectively collect information from a large pool of data. To determine the existence of a TSS at a given position, each attention head assigns scores to 512 upstream positions, determining the significance of information represented by those hidden states. The average and maximum scores at each position (Supplementary Figures S1 and S2) reveal that the majority of attention heads is highly selective w.r.t. the location from which they use information. In other words, the majority of attention heads are exclusively focused on certain promoter regions.

As explained in Material and Methods, the attention scores are calculated through the multiplication of the $\boldsymbol{q}$ and $\boldsymbol{k}$ vector, each derived from $\boldsymbol{h}$. The hidden state incorporates information about the nucleotide sequence, its positioning (i.e. nucleotide distance) with respect to the other hidden states, and any information added by the attention heads of the previous layers. Based on the distribution of the attention scores along the upstream positions, it is clear that the model is able to differentiate between these two types of information embedded within $\boldsymbol{q}$ and $\boldsymbol{k}$.

### Attention heads show to specialize towards detecting transcription factor-binding sites

The majority of attention heads that have a high-spacial selectivity is delimited in the region spanning from –100 to +20 with respect to the TSS (Supplementary Figures S3 and S4). Note that, through the presence of a convolutional layer in the attention heads, the $\boldsymbol{q}$ and $\boldsymbol{k}$ vectors are derived from the seven neighboring hidden states centered at each position (see Material and Methods). As such, the information acquired by the attention head at a given position constitutes the 7-mer centered at that position.

**Figure 3:**
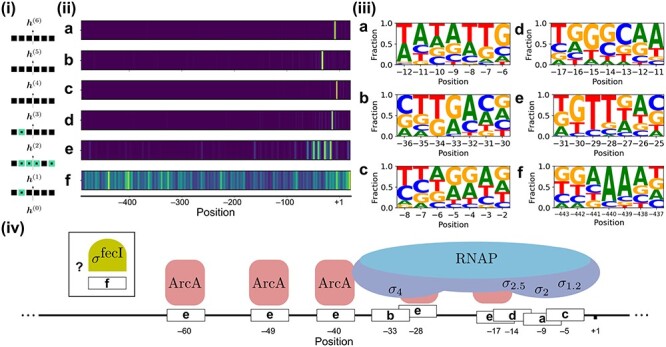
Examples of information extracted from the trained transformer network. **(i)** An illustration of the attention heads (squares) used to calculate the intermediary hidden states $\boldsymbol{h}$ within the model architecture. There are six attention heads for each layer. The green squares show the positions of the attention heads described here. **(ii)** For the six discussed attention heads, attention scores given to each of the 512 upstream hidden states are shown, averaged for 18 295 random positions on the genome (1% of the test set). Several attention heads are highly targeted based on positional information, showing high scores in yellow and low in blue. The scores are normalized for each attention head. **(iii)** For every attention head, sequence motifs were made based on the 50 highest scoring nucleotide sequences at the upstream position with the highest average score, illustrating the sequence information that returns high scores for each attention head. **(iv)** Based on the sequence and positional information from (ii) and (iii), a match exists that connects the focus of attention heads to the workings of known transcription factors involved in the transcription process.

For each attention head, sequence motifs were created based on the 50 highest scoring samples at the position with the highest averaged score (Supplementary Figures S5 and S6). These sequence motifs reflect, in addition to the positional information, the characteristics of the sequence that result in high attention scores. For example, some of the attention heads in the 2nd layer of the network are identical with the binding boxes of RNA polymerase. Here, the 1st and 2nd attention head focus on nucleotide bases ranging from approximately –12 to –6, which interact with region 2 of several $\sigma $ transcription factors. These make up parts of the RNA polymerase holoenzyme as it forms the open complex [28]. The motif obtained through selection of the highest scoring sequences is analogous to the reported consensus sequence (TATAAT) of the constitutive $\sigma ^{70}$ factor (Figure 3A). Another attention head overlaps with the –35 box, to which region 4 of the $\sigma ^{70}$ transcription factor [33] binds. The obtained sequence motif (Figure 3B) is again analogous to the reported consensus sequence (TTGACA). Nucleotides at the –6 and –5 sites have affinity to $\sigma _{1.2}$ [14], captured by another attention head. Feklistov $et$$al$. [10] report the presence of GGGA downstream of –10 motif to allow transcription even without the –35 element (Figure 3C). Barne $et$$al$. describes the interaction of region $\sigma _{2.5}$ of the RNA polymerase enzyme with the extended –10 promoter region (TGn) [4], spanning the positions –15 to –12. Alternatively, the positioning and obtained sequence motif of a potentially related attention head (Figure 3D) show resemblance with the –10 element of the $\sigma ^{54}$ transcription factor (TTGCAA) [5]. The properties of another attention head show high similarities with the binding boxes and motifs of Aerobic respiration control protein (ArcA). There have been 76 identified binding sites for ArcA in *E. coli*. The majority of these detected binding sites are situated within the region spanning from –50 to +1. The binding site consensus sequence is the repeat element (TGTTAA), featuring a distance of 11 base pairs [25]. The 2nd and 3rd element (GT) are the most conserved, similar to the constructed sequence motif (Figure 3E). In general, attention heads that do not show to be delimited to certain locations but show strong sequence motifs can constitute transcription factors with varying binding sites within the promoter region. For example, Figure 3F gives the characteristics of an attention head that has high similarity with the binding motif of $\sigma ^{\textrm{fecI}}$ (GGAAAT).

Due to the complexity of the subject and the vast amount of literature, it is difficult to verify the function of attention heads without doing extensive research for all potential elements. In addition, not all mechanisms related to the transcription process are identified. Attention head 1 of layer 6 is highly specific for position –82, which is generally associated with the binding box of the Lac Repressor [12]. Alternatively, the –82 site has been reported to overlap with other transcription factor binding sites, such as the helix-turn-helix-type transcriptional regulator IscR protein [30] and the Cyclic adenosine monophosphate receptor protein [16].

### The influence of the attention heads on the model can be mapped

To further investigate the importance of each attention head with respect to the final model prediction, two strategies were applied. First, the correlation between the attention scores at each position with the model output is quantified using the Spearman’s rank correlation (Supplementary Figure S7). As the hidden state $\boldsymbol{h}$ passes through multiple layers ($\boldsymbol{h}^{(0)} \rightarrow \ldots \rightarrow \boldsymbol{h}^{(k)}$), it is expected that the information embedded within the intermediary hidden states gradually reflects the model output. We assume this to be true, as the maximum and/or minimum correlation coefficient at any position increases gradually in the first three layers. The results indicate that the attention heads of the first two layers have the largest impact on the model output. The attention heads specialized towards several important promoter regions, as discussed in the previous section, are present in the 1st and 2nd layer.

A second strategy can compare the distribution of attention scores of two groups of samples, those that result both in the highest and lowest model predictions towards the presence of a TSS. An initial analysis highlights the importance of the attention heads that are specialized towards detecting $\sigma $ factor binding boxes, showing a large difference between the distribution of attention scores at these positions (Supplementary Figures S3 and S4).

### The output probabilities of the model reflect a correlation between sense and antisense

The output of the transformer-based model results in a continuous output probability profile along the genome and is unlike previous studies [21, 27, 39]. Evaluation of the output profile has offered further insights into the workings of the model and the related biological process. Similar to previous sections, the results are obtained from the transformer network trained on the curated annotations.

**Figure 4:**
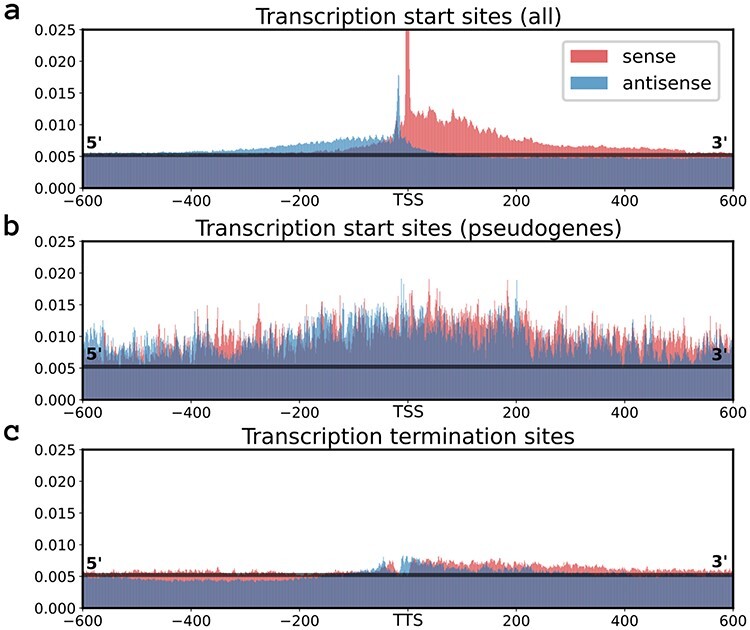
An overview of several plots that describe the prediction characteristics of the model. Data is obtained by the model trained and evaluated on the test set of the curated annotations. the median model output on the test set (0.053) is represented as a horizontal black line in (a,b,c). Each plot gives the median model output at each position within a 1201-nucleotide window centered on the site of interest. Displayed are the median model outputs for the positions around all the (**a**) Transcription Start Sites (TSSs), (**b**) the TSSs of genes annotated as pseudogenes and (**c**) all the transcription termination sites, obtained from Ju $et$$al$. [18].

Increased model outputs are generally concurring on both the forward and reverse strand. Figure 4A shows the median values at each position around the TSSs. This shows the increased output probability on both the sense and antisense strand, where the latter does not only show overall increased activity, but also peaks upstream of the TSSs. Analysis of the probability profile reveals an overall increased output probability for regions with no coding sequence present on either strand. The median output probability over the full region of the test set equals 0.0052. The median probability over all nucleotide positions with a coding sequence present on either strand is 0.0049, a value in line with the median over the full test set. The median value is more than doubled (0.0105) for nucleotides situated in between coding sequences. A higher probability for antisense is not only present for bordering and outward-facing coding sequences, but also as a higher model output on both strands is prevalent for bordering coding sequences within operons. Using the operon mapping featured on RegulonDB, the median probability within the noncoding sequences of operons is 0.0079 for sense and 0.0071 for antisense. These short sequences have a large number of peaks, where the median over the maxima of each region is ca. 10 times the baseline with 0.0415 and 0.0480 for sense and antisense, respectively.

Transcription termination sites were obtained from Ju $et$$al$. [18] and shown in Figure 4C. The median scores show a slightly increased probability score around the transcription termination sites for both strands with a shifted dip at the center. Lastly, pseudogenes were found to encompass areas with a substantial increase of output probability along both coding and noncoding nucleotide sequences, resulting in a high number of false positives. The median output probability within the coding sequences of pseudogenes is 0.0111. Most interestingly, the profile around these regions is unique, as no patterns can be discerned. Figure 4B gives the median values for each position around the TSSs of coding sequences annotated as pseudogenes. Supplementary Figure S11C gives an example of a pseudogene region.

Supplementary Figure S11 shows the model output along the curated TSSs annotations as displayed on the UCSC browser (Supplementary Data). The figure shows examples of the close interaction between the sense and antisense of the model output. The mapped reads of the SMRT-Cappable-seq experiment are also displayed.

## Discussion

The advancements in the field of functional genomics and deep learning offer new opportunities towards gaining understanding of biological processes. Today, several methods exist that analyze the sensitivity of the input features with the model output and are applicable irrespective of the model architecture [1, 45]. However, existing methods can be limiting when the biological process driving the annotation task involves complex mechanisms. These can, for example, involve overlapping binding regions. To obtain a more layered understanding into the decision-making process of trained models, methods can be created that are bound to the model architecture itself. Gaining insight into deep learning models might therefore require the coordinated effort of model design and specialized techniques that seek to explain their functioning.

In the case of functional genomics, the training of machine learning models on genome annotation tasks generally raises several challenges: (i) the imbalance between output classes, a negative set, which is several orders of magnitude larger than the positive set; (ii) the lack of high-quality annotations, where a high amount of noise is present; (iii) the dimensions of the input feature space, a multi-million length nucleotide sequence; and (iv) the biological variation of the annotation landscape, which often depends on, among other factors, the (sub)-species, the growth phase and the growth conditions of the organism.

We recently introduced a transformer architecture specialized for processing full genome sequences that proved to be well equipped to address the issue of class imbalance and dimensionality of the input space. State-of-the-art performances were returned on the annotation of TSSs, translation initiation sites and methylation sites [6]. Following research suggesting that attention heads mapping semantic relatedness between words [38], we have investigated whether the attention heads of the transformer-based model for genome annotation tasks can be leveraged for information. With over 250 identified potential factors involved in the transcription process of *E. coli* [23], the detection of TSSs serves as a perfect case study to delve into the decision-making process of trained models.

A curated set of TSSs was created and was shown to result in better performances than any individual set of annotations available. Potentially, the selection of TSSs from multiple data sets can prevent the model to fit on systematic noise of individual data sets, and make way with missing annotations. Discrepancies occurring between the different annotations might point to an influence of growth conditions on the expression profile. Given that the *in vivo* expression profiles of Cappable-seq, SMRT-Cappable-seq and SEnd-seq were obtained from *E. coli* under optimal growth conditions, the detection of TSSs activated by specialized $\sigma $-factors, such as $\sigma ^{38}$ for stress responses, $\sigma ^{32}$ for heat shock response and $\sigma ^{28}$ associated with flagellar genes [37], are bound to be limited. We have furthermore observed that the incorporated methods do not provide a precision at single-nucleotide resolution (Figure 2), potentially leading to the multi-nucleotide peaks observed for both the attention heads (Supplementary Figures S3 and S4) and model output (Figure 4). The higher number of shifts in the annotations from RegulonDB indicate this data set to be of lower quality. Today, investigation of TSSs or promoter sequences is generally performed using these annotations [21, 27, 39], a choice that might not be justified.

The shifts in annotated TSSs might also be the result of a transcription process that is not fixed and allows a more flexible start site for 5*′* untranslated regions. To evaluate the impact of these multi-nucleotide peaks on the reported PR AUC scores, we averaged the output probabilities over larger $k$-mers and labeled these positions positive if a TSS is present. After reducing the annotation resolution to 4-mers, the PR AUC score is more than doubled (0.29). This score stabilizes when the resolution is even further reduced, e.g. 10-mers result in a PR AUC of 0.33.

Our findings establish the specialization of the attention heads, which reveals new opportunities towards explainable deep learning in this field. Focusing on the function of individual attention heads by evaluating the attention scores given to the upstream hidden states, we were able to characterize its functioning by answering a variety of questions, such as ‘From which positions within the promoter region does the attention head generally extract its information?’ and ‘What is the optimal nucleotide composition to obtain the best attention scores?’. Based on this information, we identified attention heads specialized towards well-known transcription factors (Figure 3) and related their importance towards the model output by comparing the scores of the best and least scoring samples for these attention heads (Supplementary Figures S5 and S6). In this study, evaluations are based on the positions with the highest average attention scores and have therefore given less focus towards attention heads that are not highly targeted spatially.

We were able to characterize many important elements related to the biological process the model was trained for. As shown, the effectiveness of the transformer model to specialize certain attention heads towards identifying important promoter regions is enhanced by the convolutional layer that incorporates information from seven neighboring hidden states. This length works well for the binding boxes of the $\sigma ^{70}$ factor, featuring a motif length of six nucleotides, but would logically not be optimal for transcription factors that involve binding regions that are larger than 7 nucleotides (e.g. LacR, ArcA). Although many of the characterized attention heads (Supplementary Figures S5 and S6) have not been linked to existing transcription factors, further studies on the topic are likely to uncover more matches between the function of attention heads and the transcription initiation process.

In addition to characterizing the individual attention heads, future approaches can be outlined that aim to map the relatedness between these attention heads and the model output. Higher-order interactions of the attention score profiles between different attention heads might uncover another source of information that offers understanding towards the biological process. Attention heads can, for example, be correlated in order to map transcription factors with binding sites larger than 7 nucleotides. Independent mechanisms for transcription might also be revealed this way. For example, this might be applicable for attention heads picking up alternative $\sigma $ factor-binding sites, the –6∖–5 motif allowing transcription without the –35 element [10] or the extended –10 element allowing transcription without the –10 element [4]. Supplementary Figure S10 shows the correlation matrix between the attention scores of the different attention heads.

In a previous study, it was urged that attention scores do not necessarily offer transparency into the neural network, as only a weak relation between attention scores and feature importance was found on natural language processing tasks [17]. Similarly, specialization of the attention heads cannot be ascertained for other prediction tasks or model architectures and will have to be evaluated on a case-by-case study. Nonetheless, we believe that it is likely that similar observations will be made for other annotation tasks, as no manual tweaking to the model architecture or prediction task was performed in preparation of our observations. In addition, the specialization of attention heads, which is able to combine both positional and sequential information, is similar to the specialization of kernels towards motifs or shapes in a convolutional neural network, which is widely accepted to occur for models trained on nucleotide sequences or images.

Processing of the full genome is unique for this type of study, as the negative set on which the model is trained is normally subsampled [21, 27, 39]. The presence of TSSs (and many other genomic sites of interest) for neighboring positions is calculated from largely the same information, yet is expected to return a distinct output profile. The availability of the model predictions along the full genome sequence might result in the identification of model flaws or properties on the transcription process that would not be retrieved otherwise.

The increased probabilities along the sense and antisense of noncoding regions corroborate mechanisms of interference as described in literature [31]. Several types of transcription interference exist that involve convergent promoters: promoter competition, sitting duck, occlusion, collision and roadblock interference. Both sitting duck and roadblock interference involve the occurrence of a RNA polymerase open complex that is too weak or strong for promoter escape. Promoter competition involves the inhibition of bordering promoters, whereas occlusion and collision are related to the inference of head-on elongating RNA polymerase. Recently, through the analysis of the SEnd-seq data, the occurrence of head-on interference is described to be an under-appreciated mechanism of transcription regulation [18], an explanation that fits well with neighboring peaks of the model output on the sense and antisense of the genome. At this point, RNA polymerase-binding site that do not result in RNA are likely contributor to false positive predictions. These include regions where transcription initiation is blocked, such as is the case with head-on interference or sequences where promoter escape is impossible. Head-on interference, among other scenarios, might furthermore result in RNAs that are too small for detection by *in vivo* methodologies. To further improve model performances on TSSs, possible options include the incorporation of both sense and antisense up– and downstream of the evaluated position.

The low-transcriptional activity common for pseudogenes [26] could be explained by the interfering affinity of the RNA polymerase for both sense and antisense, as indicated by the probability profile of the model output (Figure 4B and Supplementary Figure S11C). Even though the existence of pseudogenes is not fully understood, it is characterized by an overall higher probability output of the model on both sense and antisense, potentially indicating a high activity of head-on interference.

To conclude, the study reveals that the attention mechanism of the transformer-based models offers new opportunities for explainable deep learning in genomics. The availability of both sequence and positional information towards determining attention scores reveals the influence of both factors. Combined with the observation that attention heads perform distinct functions, we were able to determine both the motif and position related to several essential factors involved in the process. In addition, this property might result in correlation studies between the attention scores of different attention heads, potentially unveiling the relatedness between regulatory elements or mechanisms. Using all this information, it is possible that further analysis might be aided by *in vivo* validation methods, thereby fueling the discovery of new mechanisms.

**Key Points**

- In this work, we focus on exploring advantages that transformer networks could provide towards interpretable machine learning for genome annotation tasks.
- For detection of TSSs, we are able to characterize the function of attention heads within the model and find these to be specialized towards the detection of transcription factor-binding sites. Without any manual input, we can pinpoint the location of several essential promoter elements, their optimal sequence motifs and their importance.
- Although we provide this analysis for TSSs specifically, the methods outlined are generic and applicable for any genome annotation task.

## Supplementary Material

supplementary_files_bbab060

## Data Availability

All models and code used are available on GitHub (https://github.com/jdcla/DNA-transformer). Supplementary File 1 features the full technical details of the transformer network and supplementary figures. The annotations and model output are given in Supplementary File 2. The annotations and model outputs of the test set of the curated annotations can be browsed on UCSC (https://kermit.ugent.be/files/UCSC/UCSC_browser.html).

## Notes

**Dr Jim Clauwaert** recently obtained his PhD at Ghent University under supervision of Willem Waegeman and Gerben Menschaert. His PhD thesis was titled ‘Deep learning techniques for genome processing and annotation tasks in prokaryotes’.

**Prof. Gerben Menschaert** is the CSO and co-founder of OHMX.bio. He also holds a position as guest professor at Ghent University. He is an expert in high-throughput technologies (sequencing and mass spectrometry) and bioinformatics.

**Prof. Willem Waegeman** is an associate professor at Ghent University. He is a machine learning expert with interests in method development and challenging applications in the life sciences.
