# Supplementary material for: Explainability in transformer models for functional genomics
Source: Brief Bioinform. 2021 Apr 8;22(5):bbab060. doi: 10.1093/bib/bbab060 (PMC8425421; doi:10.1093/bib/bbab060)
Supplement: supplementary_files_bbab060 [file supplementary_files_bbab060.zip › authors_biography.docx]

**Authors biography**

**Dr. Jim Clauwaert** recently obtained his PhD at Ghent University under supervision of Willem Waegeman and Gerben Menschaert. His PhD thesis was titled “*Deep learning techniques for genome processing and annotation tasks in prokaryotes*”.

**Prof. Gerben Menschaert** is the CSO and co-founder of OHMX.bio. He also holds a position as guest professor at Ghent University. He is an expert in high-throughput technologies (sequencing and mass spectrometry) and bioinformatics.

**Prof. Willem Waegeman** is an associate professor at Ghent University. He is a machine learning expert with interests in method development and challenging applications in the life sciences.
