# Supplementary material for: Explainability in transformer models for functional genomics
Source: Brief Bioinform. 2021 Apr 8;22(5):bbab060. doi: 10.1093/bib/bbab060 (PMC8425421; doi:10.1093/bib/bbab060)
Supplement: supplementary_files_bbab060 [file Rebuttal_BiB (1).pdf › Rebuttal_BiB (1).pdf]

# Author rebuttal

We would like to thank the reviewers for their valuable feedback. In this rebuttal, we provide a point-by-point response to the comments of the reviewers. Our manuscript changed significantly. Therefore, a version with track-changes is provided in a separate pdf-file, in addition to the revised manuscript. Remark that the title of the article has changed a bit.

**Reviewer 1**

**In this paper, the authors proposed a transformer-based neural network model for the detection of transcription start sites applied on E. coli. The major claimed contributions are summarized as follows:**

**(1) The authors created an ensemble set of transcription start sites by aggregating four different datasets.**

**(2) The authors used a byproduct of the transformer network, the attention, to highlight the genomic annotation from a interpretability perspective.**

**1. What's the motivation of introducing segment in the neural network model?**

The segment is a necessary result of the model not being able to process the full input sequence at once. Separating the input sequence into shorter sequence lengths allows the processing of a full genome in a timely manner. In this paper, the size of the segment length is identical to the one applied in our previous article, where a length of 512 proved to work well [1]. The most important factor when determining the segment length is related to the window over which attention is calculated. Reducing the segment length under 100nt drastically reduces the model performance. Changing the segment length between ~300-1000 does not really impact the performance, and only influences training times, as this influences the size of the calculated attention matrices. A more in-depth evaluation of the varying segment lengths is given in our previous work.

[1] https://ieeexplore.ieee.org/document/9246292

**2. Is the segment robust to the genome base-pair shift? For example, in the paper, 1st bp - 512th bp correspond to the first segment, and 513th bp - 1024th bp correspond to the second segment, and so on. What if there is some deletion happened at the first bp, and as the consequence, 2nd - 513th bp correspond to the first segment, and 514th - 1025th bp correspond to the second segment, and so on. The learned hidden state should differ drastically, right? More detailed robustness evaluation is needed.**

The positional information processed by the model is not absolute. When evaluating the presence of a transcription start site at position *n*, the method processes the 512 hidden states containing information of the nucleotide sequence and the states' relative positioning to *n*. Additionally, hidden states of the previous segment are made accessible to ensure the model has access to the 512 previous hidden states, independent of the positioning of *n* within the segment. Varying the nucleotide segments created from the input sequence does not have any effect on the information processed by the model and therefore its prediction.

It is only during training that the use of non-overlapping segments (0-512, 513-1024, …) might have a negative influence. As the gradient cannot be propagated back through the hidden states of the previous segment, optimization of the model weights is only performed based on partial derivatives obtained within the current segment. As such, for a prediction on the nucleotide that is positioned first in a segment, no gradient can be attained on the hidden states from the 511 hidden states obtained from the previous segment. Potential effects of this have been evaluated in more detail in our previous work. We have not observed any negative effects.

In practice, as one of the many parameters that regulates the training process of a transformer-XL model, the segment length is varied for every batch between a length of 512nt - 51nt and 512nt + 51nt (+-10%). Thus, the absolute positioning of the nucleotides within each segment varies each epoch, and might be a contributing element to the observation that no negative effects are reported from the fact that gradients cannot be backpropagated through the cached hidden states of the previous segment.

Although important to understand, we have opted to exclude the explanation of core functionalities of the transformer-XL architecture from the main manuscript. A more in-depth explanation is given in the supplementary files. We believe that it suffices to refer to the original transformer-XL article and our previous work for an in-depth discussion. Including more details in the paper would negatively impact the size and focus.

**3. The validity of applying attention for explanation is still questionable (Jain Sarthak and Byron C). The authors need provide convincing reason why the genomic annotation is immune to the common criticism raised.**

We thank the reviewer for the provided reference and have included a short discussion on the matter in the paper (discussion). Jain Sarthak and Byron C. Wallace show that the attribution of attention scores does not necessarily correlate to feature importance of the input w.r.t. the output. As such, they urge caution with the claim that networks that apply attention offer more transparency. We understand the study to be of considerable interest to the community, and don't see any conflicts with our submission.

Essentially, we do not claim, but rather find that the attention scores/distributions provide some transparency about the working of the model or the biological process that drives the annotation it was optimized for. We analyze the characteristics of attention heads, of which we could easily obtain observations that are valuable for discussion. As is discussed in the article, this pertains to the function of the attention heads and the molecular process behind the prediction task. We found attention heads that are specialized towards specific transcription factors, and were able to characterize these by evaluating the positional and nucleotide information resulting in high attention scores. We validate these to be binding sites and motifs of well-known transcription factors based on information from previous studies.

Based on these findings, we believe there is sufficient material to warrant a discussion on the topic. We believe this research to be relevant, as it is unlikely that the discussed observations would only occur for this prediction task.

**4. The paper is very poor-written and difficult to follow.**

We thank the reviewer for the feedback. We have now invested further in readability, grammar and general construction of the manuscript. We additionally iterated over the paper several times in an attempt to improve the reading experience. In case the reviewer has further suggestions for improving the paper, we are willing to take those into account.

## Reviewer 2

**In “Explainable transformer models for functional Genomics in Prokaryotes”, Clauwaert and colleagues describe the use of transformers to predict TSS positions on E coli genome. This work is very similar to an earlier work published both in BioRxvi and in IEEE/ACM Transactions on Computational Biology and Bioinformatics. In the present paper, the authors present new results on alternative data-sets for TSS positions as well as a biological interpretation of the trained network. Overall, the major interest of the paper in my eyes is to popularize the use of transformers for the bio-informatics community. As such, it only partially meets its goal. I have organized my review in major and minor points to address:**

With this submission, we address the following research questions, as outlined by the first reviewer:

(1) Assess the application of attention towards gaining insights into the decision-making process of the model, increasing our understanding of the model and the biological process that drives the presence of a genomic site of interest.

(2) In order to achieve (1), we seek to improve the quality of the annotated data sets by leveraging data obtained through multiple methodologies. We give a short overview of our findings. This is of interest, as today, no data set exists that represents the ground truth.

We believe that these research questions are very different from our previous publication, which focused on:

(1) Introducing a new network architecture (transformer-XL) for genome annotation tasks. Evaluate the advantages/disadvantages this model architecture offers as compared to existing methodologies applied for genomics.

(2) Optimizing this model architecture towards processing nucleotide sequences (instead of sentences).

(3) Creating a benchmark to compare the performances of the new network architecture with previous methodologies.

We have iterated over the full article to make sure that the differences in focus with our previous work are clear.

### Major Points:

**1) There are parts of the article that are copied and paste from their previous work. While this may be normal for a computer scientist community, it is not acceptable for the bio-informatics community.**

We have iterated over the article to remove any redundancy with the published version of the IEEE article[1].

**2) The corollary of point 1 is that the model should be simply explained to an audience that may not know deep learning techniques. I would recommend adding a schematics explaining the differences between CNN, RNN and transformer models used in the field as well as a more detailed discussion of pros and cons.**

To address this issue we have implemented a short discussion and table in the supplementary files containing the best known architectural structures in deep learning, their applications in the bioinformatics field, their strengths and computational demands. We have also invested into improving the text explaining the workings of the transformer model.

The flexibility of deep learning architectures has been key in offering an answer to a variety of prediction task problems. Deep learning has thereby become a complex field, featuring complex architectures. We have been unable to expand upon the architectural structure of various networks and their advantages/disadvantages in function of specific settings. This was not possible using the limited amount of space available. In order to guide the reader, we have included references within the manuscript towards reviews that were specifically created to achieve this.

**3) The network application to E coli TSS is interesting but was already in the first paper. It would be very valuable to add other data-sets from other species, possibly human.**

In our previous publication [1], we have outlined the advantages of the model architecture and have evaluated its performance with other methods. In this work, we can state the efficacy of the method without requiring additional benchmarking results. TSSs and the detection thereof has enjoyed considerable importance towards the scientific community. For these reasons we believe that the further evaluation of the transformer network trained specifically for the detection of TSSs to be the right choice.

Due to the distinct differences between the research objectives of our previous publication and this submission, we see it to be an important advantage to apply the model, as described in a previous work, in this setting. As such, we can build upon our previous work without the need to include information that is not related to the objective of this study.

Using the current network, it is not possible to process the full genome sequence of humans. This is a limitation of the computational requirements, as a human genome constitutes a nucleotide sequence that is ca. 1000 times larger than the prokaryotic genome. Furthermore, other molecular mechanisms, such as RNA splicing, make this task more complicated for eukaryotic genomes.

**4) The author need to deal with the class imbalance problem. This has also been explored in the context of TSS predictions in Khodabandelou et al., PeerJ computer science 2020. It would be valuable for the authors to explain how they deal with the problem. Do they use different weights for positive and negative class examples ? Can they compare the transformer performances with the CNN approach described in the Khodabandelou et al paper ?**

As the reviewer rightfully points out, class imbalance is the norm for genome annotation tasks, and can negatively influence the performance of the model if not properly addressed. Today, the two most common strategies are: (1) subsampling of the negative sets, (2) weighing the loss function. Typically, applying one of these strategies results in higher performances of the trained model.

As the full genome is processed, subsampling of the negative set is not possible. During evaluation of varying model architectures, as part of our previous publication, it was observed that weighing the loss function did not influence the final performance of the trained model (although it resulted in different optimization curves). We are not sure why the existing class imbalance does not seem to influence the performance of the trained model on transformer architectures, but don’t believe this to be raised as an issue, as the model was shown to outperform previous methods.

Essentially, the issue only pertains to the performance and evaluation of the model. With regard to the performance, we believe the discussion of other potentially viable strategies to deal with class imbalance to be outside of the scope of this paper, and are not in need of any strategies that have not been previously described. For validation, we apply the ROC AUC and PR AUC precisely for these reasons. As described in the article, the PR AUC reveals the weakness of the model (or any other model performing this task today) when applied on the genome, where the precision and recall of the top-k predictions are of practical interest.

Comparison of the transformer model with the article of Khodabandelou et al. is not possible, as it is currently not feasible to apply the transformer-based architecture for human genomes (see previous comment). Additionally, the study of TSSs in eukaryotes, or more specifically humans, is outside of our expertise and would require a vast amount of research on our side to make sure the model architecture is suited given the challenges of this prediction task.

**Minor points:**

**5) On the fig 2a can the authors check the numbers (esp. 11717) in the venn diagram ?**

After evaluation of the Venn diagram we can confirm that the numbers are correct. The high amount of instances is in accordance to the high amount of annotated TSS in the dataset obtained through Cappable-seq (See Table 1).

**6) On fig 2b it may be easier to read the histograms if the authors would only show one annotation against every other annotation of each panel. Ie. they could remove the blue bars from the first panel, on so on.**

Within a single data set, it also occurs that TSSs are annotated in close vicinity (i.e. <5nt). We believe that it is important to also include this information. In order to better communicate the data shown in the graphic, we have improved the explanation given by the caption.

**7) The two last paragraph of “Related works” could be integrated together as the mention of DeepLIFT (for instance) is redundant.**

We have adjusted the article as suggested by the reviewer, and have reduced the related paragraphs as such.

**8) the authors may wish to remove their citation to Kuo-Chen Chou who is a renown scientific impostor : https://www.nature.com/articles/d41586-020-00335-7**

We have adjusted the article as suggested by the reviewer.

[1] https://ieeexplore.ieee.org/document/9246292
